# Heme Oxygenase 1 and 2 Common Genetic Variants and Risk for Essential Tremor

**DOI:** 10.1097/MD.0000000000000968

**Published:** 2015-06-19

**Authors:** Pedro Ayuso, José A.G. Agúndez, Hortensia Alonso-Navarro, Carmen Martínez, Julián Benito-León, Sara Ortega-Cubero, Oswaldo Lorenzo-Betancor, Pau Pastor, Tomás López-Alburquerque, Elena García-Martín, Félix J. Jiménez-Jiménez

**Affiliations:** From the Department of Pharmacology (PA, JAGA, CM, EG-M), Universidad de Extremadura, Cáceres; Research Network on Adverse Reactions to Allergens and Drugs (PA, JAGA, CM, EG-M); Department of Medicine-Neurology (HA-N, FJJ-J), Hospital “Príncipe de Asturias,” Universidad de Alcalá, Alcalá de Henares; Section of Neurology (HA-N, FJJ-J), Hospital Universitario del Sureste, Arganda del Rey; Service of Neurology (JB-L), Hospital Doce de Octubre, Department of Medicine, Universidad Complutense, Madrid; CIBERNED (JB-L, SO-C, OL-B, PP), Centro de Investigación Biomédica en Red de Enfermedades Neurodegenerativas, Instituto de Salud Carlos III; Neurogenetics Laboratory (SO-C, OL-B, PP), Division of Neurosciences, Center for Applied Medical Research, Universidad de Navarra; Department of Neurology (SO-C, OL-B, PP), Clínica Universitaria de Navarra, University of Navarra School of Medicine, Pamplona; Department of Neurology (PP), Hospital Universitari Mutua de Terrassa, Terrassa, Barcelona; and Department of Neurology (TL-A), Hospital Universitario de Salamanca, Spain.

## Abstract

Several reports suggested a role of heme oxygenase genes 1 and 2 (*HMOX1* and *HMOX2*) in modifying the risk to develop Parkinson disease (PD). Because essential tremor (ET) and PD share phenotypical and, probably, etiologic factors of the similarities, we analyzed whether such genes are related with the risk to develop ET.

We analyzed the distribution of allelic and genotype frequencies of the *HMOX1* rs2071746, *HMOX1* rs2071747, *HMOX2* rs2270363, and *HMOX2* rs1051308 single nucleotide polymorphisms, as well as the presence of copy number variations of these genes in 202 subjects with familial ET and 747 healthy controls.

Allelic frequencies of *rs2071746T* and *rs1051308G* were significantly lower in ET patients than in controls. None of the studied polymorphisms influenced the disease onset.

The present study suggests a weak association between *HMOX1* rs2071746 and *HMOX2* rs1051308 polymorphisms and the risk to develop ET in the Spanish population.

## INTRODUCTION

Despite the role of genetic factors in the pathogenesis of essential tremor (ET) is supported by substantial evidence, the identification of the responsible gene(s) remains to be clarified (revised in references).^1,2^

Heme oxygenase (HMOX) is an essential enzyme in heme catabolism, and it occurs as 2 main isozymes, an inducible heme oxygenase-1 (HMOX1) and a constitutive heme oxygenase-2 (HMOX2), which are encoded by the genes designated, respectively, as *HMOX1, HO-1*, or *HSP32* (gene identity 3162, chromosome 22q13.1) and *HMOX2* or *HO-2* (gene identity 3163, chromosome 16p13.3).

Recently, several studies have shown association between *HMOX* genes and the risk for Parkinson disease (PD). Although Funke et al^3^ found no association between 4 genetic markers of *HMOX1* with susceptibility for PD, other group found a synergistic association of *HMOX1* rs2077146TT genotype both with *glycogen synthase kinase 3-beta* (*GSK3beta*) gene^4^ and with pesticides exposure,^5^ increasing the risk for PD. We previously reported an association between the variable number tandem repeat of alternating purine-pyrimidine sequence (GT)_n_ and the single nucleotide polymorphism (SNP) rs2071746 in the *HMOX1* gene with the risk of developing PD, especially with early onset of the disease and with the classic PD phenotype, whereas rs2071747 and rs9282702 SNPs showed no association.^6^ With regards of the *HMOX2* gene, our group reported an increased risk for PD among rs2270363GG carriers, and lack of association with rs17884623 and rs17880805 SNPs.^7^

Because ET and PD are 2 common disorders that share many epidemiologic, genetic, clinical, neuroimaging, and neuropathological features,^8–10^ it seems reasonable to study the possible association between SNPs previously associated with PD risk, and the risk for ET. To investigate a possible association between *HMOX1* and *HMOX2* polymorphism and the risk of developing ET, we genotyped *HMOX1* and *HMOX2* SNPs in a large group of white Spanish ET patients and controls.

## METHODS

### ET Patients and Controls

The 202 patients included in the study fulfilled the diagnostic criteria for definite ET^11^ (100 men and 102 women, mean age 65.7 ± 16.1, mean age at onset of ET 48.2 ± 18.1 years), and 747 age- and sex-matched controls (379 men and 368 women, mean age 63.6 ± 14.6 years). ET patients were recruited from the Movement Disorders Units of 3 University Hospitals. Inclusion criteria, beside the diagnosis of definite ET, were the absence of other previous neurological diseases, positive family history of ET (at least 1 first-degree relative affected), and normal thyroid function. Controls were healthy unrelated age- and sex-matched white Spanish individuals who did not have tremor or other movement disorders (459 were recruited from the Clínica Universitaria de Navarra, Pamplona, Spain; and 288 were recruited from the Infanta Cristina University Hospital, Badajoz, Spain).

### Ethical Aspects

All the participants were included in the study after giving written informed consent. This study was approved by the ethics committees of the University Hospital “Príncipe de Asturias” (University of Alcalá, Alcalá de Henares, Madrid, Spain), the Infanta Cristina University Hospital (Badajoz, Spain), and Clínica Universitaria de Navarra (Pamplona, Spain). The study was conducted according to the principles expressed in the declaration of Helsinki.

### Genotyping

Two SNPs in the *HMOX1* gene and 2 polymorphisms in the *HMOX2* gene were genotyped by means of TaqMan probes. Analyses included the *HMOX1* SNP rs2071746, which is an upstream variant, *HMOX1* rs2071747, which is a missense mutation within the exon 1 of the *HMOX1* gene, the SNP rs2270363, which is a polymorphism in the regulatory region of the human *HMOX2* gene, and rs1051308 is a polymorphism in the 3’untranslated region. These SNPs were selected on the basis of expected allele frequency in white individuals and putative functional effects.^6,7^

Genotyping was performed in genomic DNA obtained from blood samples of participants and was carried out by means of TaqMan assays (Applied Biosciences Hispania, Alcobendas, Madrid, Spain), which were designed to detect the previously mentioned SNPs. Detection was carried out by real-time quantitative polymerase chain reaction in an Eppendorf RealPlex Thermocycler. The amplification conditions were the following: a denaturation time of 10 minutes at 96 °C was carried out, then 45 cycles of 92 °C 15 seconds 60 °C 90 seconds were carried out, and fluorescence was measured at the end of each cycle and at endpoint. All samples were determined in triplicate. Genotypes were assigned by means of gene identification software (RealPlex 2.0; Eppendorf) and by analysis of the reference cycle number for each fluorescence curve, calculated using the CalQPlex algorithm (Eppendorf).

Copy number variations (CNVs) were analyzed using the TaqMan copy number assays of the *HMOX1* and *HMOX2* genes, Hs00774483_cn and Hs01223070_cn, respectively. Both assays were designed to hybridize within the open reading frame within the target genes (Applied Biosciences Hispania, Alcobendas, Madrid, Spain). Amplification was carried out in an Applied Biosystems 7500 real-time thermocycler as described by the manufacturer, using RNAase P as a copy number reference assay. All reactions were carried out in quadruplicate. Results were analyzed by means of the CopyCaller Software (Applied Biosciences Hispania). According to standard procedures in CNV analyses, samples with a single copy of the corresponding gene were named as heterozygous (null/present). Because the probes were designed to detect exonic sequences, even if the rest of the gene would remain in these so-called null alleles, the translated protein would not be functional.

### Statistical Analysis

The Hardy–Weinberg equilibrium was analyzed with the PLINK software.^12^ Haplotype reconstruction was performed using the program PHASE v2.1.1.^13^ We used the default model for recombination rate variation with 1000 iterations, 500 burn-in iterations, and a thinning interval of 1. Further details are provided elsewhere.^14^ Statistical analyses were performed using the SPSS 15.0 for Windows (SPSS Inc, Chicago, IL). Intergroup comparison values were calculated by using the χ^2^ or Fisher tests when appropriate. The 95% confidence intervals were also calculated. Correction for multiple testing (Pc values) were calculated by using the False discovery rate procedure.^15^

The sample size was determined from variant allele frequencies observed in control individuals with a genetic model analyzing the frequency for carriers of the disease gene with a relative risk value 1.5 (*P* = 0.05). The statistical power for 2-tailed associations for the presence of the SNPs identified in this study (rs2071746, rs2071747, rs2270363, and rs1051308) was 95.06%, 38.51%, 92.72%, and 94.23%, respectively. Testing for heterogeneous association (homogeneity test) was analyzed by using the Breslow–Day test. The negative predictive value was calculated as d/r2 (d = number of control individuals with the risk factor absent, r2 = sum of ET patients and controls with the risk factor absent).

## RESULTS

The frequencies of the *rs2071746, rs2071747, rs2270363,* and *rs1051308* genotypes and allelic variants in ET were in Hardy–Weinberg equilibrium, both in ET patient and control groups. The frequencies of *rs2071746TT*, and *rs1051308GG* genotypes and *rs2021746T* and *rs1051308G* alleles were significantly lower in ET patients than in controls, although, after multiple test comparison analysis, only the differences for *rs2021746T* and *rs1051308G* alleles remained as significant (Table 1). The frequencies of *rs2071747* and *rs2270363* did not differ significantly between ET patient and control groups.

**TABLE 1:**
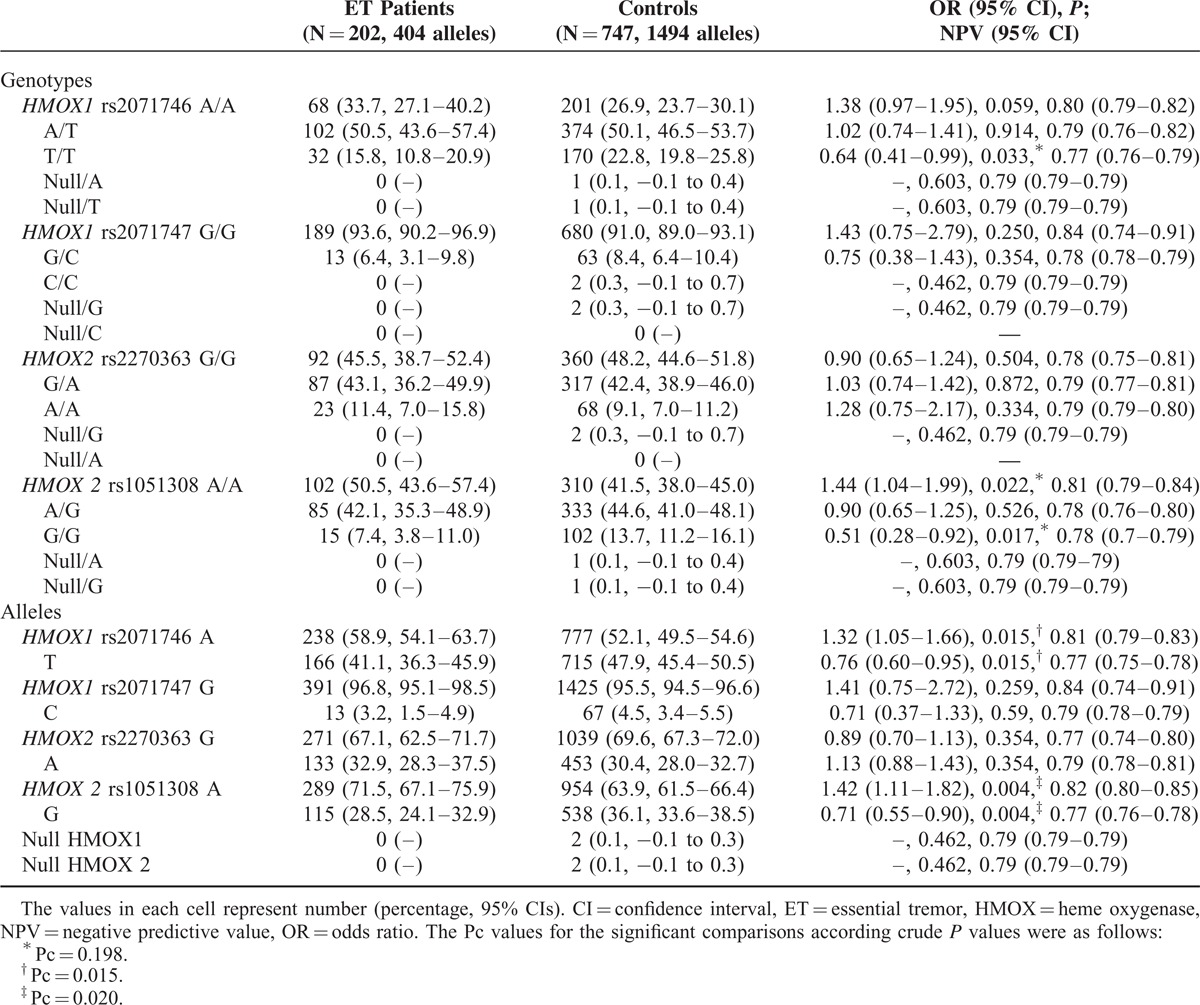
*HMOX* Genotypes and Allelic Variants of Patients With ET and Healthy Volunteers

The frequency of *rs2071746TT* genotype was significantly lower in ET men than in control men, whereas that of *rs1051308GG* genotype and *rs1051308G* allele were significantly lower in ET women than in control women; however, only the differences in *rs1051308G* allele frequency in women remained significant after multiple test correction (Table 2 ).

**TABLE 2:**
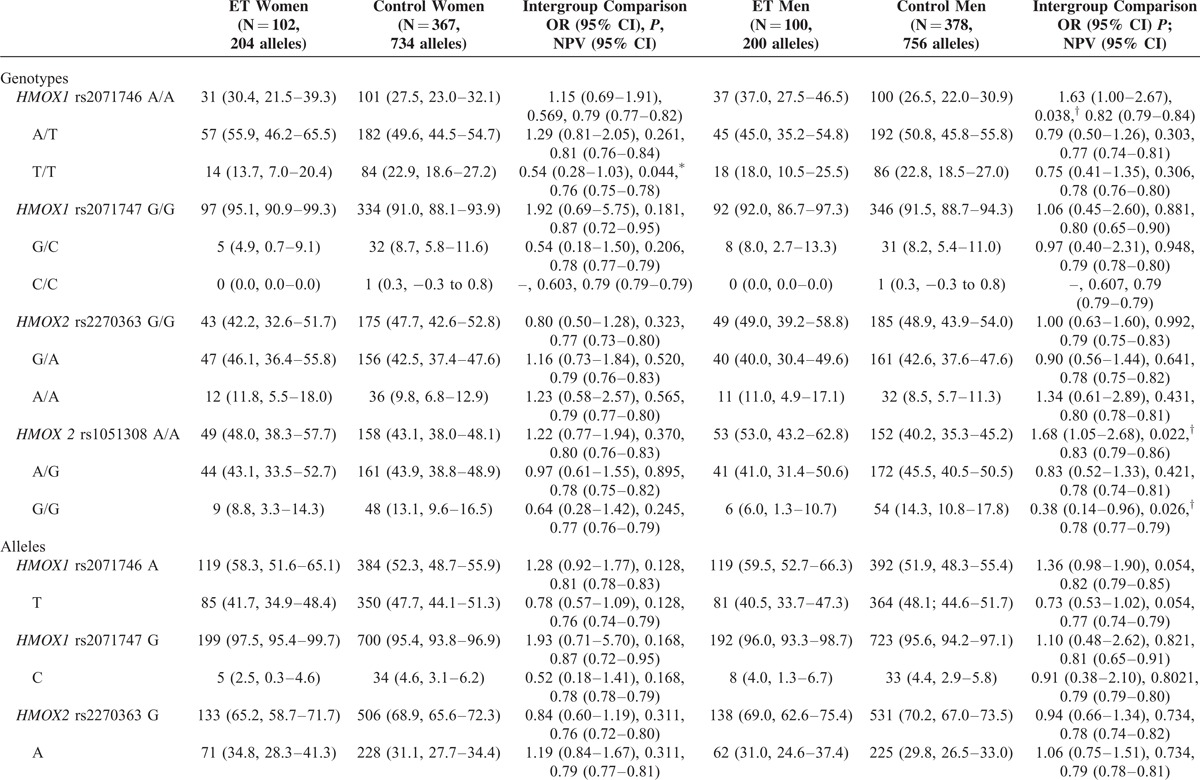
*HMOX* Genotypes and Allelic Variants of Patients With ET and Healthy Volunteers Distributed by Sex

**TABLE 2 (Continued):**
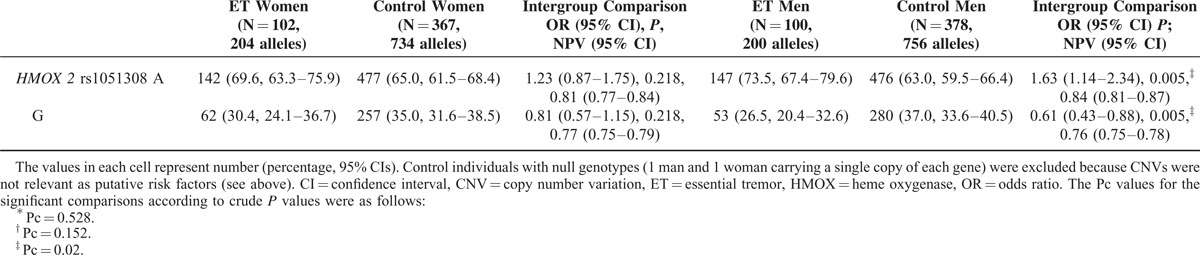
*HMOX* Genotypes and Allelic Variants of Patients With ET and Healthy Volunteers Distributed by Sex

Mean ± SD age at onset of tremor did not differ among the ET carrying *rs2071746AA, rs2071746AT,* and *rs2071746TT* genotypes (49.2 ± 23.9, 47.3 ± 26.2, and 47.6 ± 17.3 years, respectively); *rs2071747GG* and *rs2071747GC* genotypes (48.1 ± 26.5 and 45.5 ± 11.2 years, respectively); *rs2270363GG, rs2270363GA,* and *rs2270363AA* genotypes (46.6 ± 25.6, 49.8 ± 25.3, and 47.0 ± 15.2 years, respectively); and *rs1051308AA, rs1051308AG,* and *rs1051308AA* genotypes (47.6 ± 26.7; 49.4 ± 24.9, and 44.6 ± 13.0 years, respectively).

The frequencies of the *rs2071746, rs2071747, rs2270363,* and *rs1051308* genotypes and allelic variants in ET patients with head (n = 45), voice (n = 45), tongue (n = 16), and chin tremor (n = 11) did not differ significantly from those found in the control group, after correcting for multiple comparison analysis (data not shown).

CNV analyses revealed the occurrence of 2 control individuals with a single copy of *HMOX1* and another 2 individuals with a single copy of *HMOX2.* No CNVs were identified among ET group (Table 1) Individuals with 0 or ≥2 gene copies were not identified in the whole study group.

## DISCUSSION

Data from the present study suggest a weak association between the allelic variants *HMOX1 rs2071746T* and *HMOX2 rs1051308G* and the risk for ET. Previous studies reported also association between these *HMOX* polymorphisms and PD.^6,7^ However, the possible putative mechanisms suggesting an association between HMOX and ET should be considered as speculative. In a previous study, we reported association between a HMOX1 microsatellite (GT)n polymorphism and the risk of developing PD.^6^ Such a microsatellite polymorphism could not be analyzed in the present study because of DNA shortage. CNVs analyses revealed that CNV variations occur rarely in ET patients and that these gene variations do not seem to play a major role regarding risk association.

In the brain, the HMOX pathway is very important as a defensive mechanism for neurons exposed to oxidative stress, contributing to the degradation of heme to biliverdin, free iron, and carbon monoxide; and particularly HMOX1 expression has been found upregulated in the brains of patients with PD, Alzheimer disease, and multiple sclerosis.^16,17^ Moreover, upregulation of HMOX1 in astrocytes increases neuronal oxidative stress and sequestration of iron nonlinked to transferrin in the mitochondrial department.^17^

Despite the predominant role of genetic factors in the etiology of ET,^1,2,18–22^ the role of environmental factors alone or interacting with genetic factors has been also suggested.^22–24^ Several years ago, our group conducted a case–control study on the exposure to some environmental factors such as substances containing lead, mercury, manganese, solvents, and β-carbolines (toxins that produce tremor), and exposure to agricultural work, well water, pesticides, and cigarette smoking and alcohol drinking habits in patients with ET compared with controls. After a multivariate study, exposure to agricultural work and frosted glass, were significantly associated to the risk for ET, whereas age at onset of ET was significantly higher in patients exposed to iron–manganese alloys.^22^

Blood harmane (a potent tremor-inducing β-carboline alkaloid, which shares structural similarity with 1-methyl-4-phenyl-1,2,3,6-tetrahydropyridine, a neurotoxin responsible for development of one of the main animal models or PD) levels have been found increased in patients with ET.^25,26^ In addition, a recent postmortem study described increased cerebellum harmane levels in ET patients compared with controls as well.^27^ Lead levels have also been found increased in ET patients compared with controls^28,29^ and were related with the risk for ET in interaction with an δ-amino-levulinic acid dehydratase gene polymorphism.^30^ Exposures to nutritional antioxidant intake in the current diet were similar for ET patients and controls in a single study regarding this issue.^31^

The pathophysiology and neuropathology of ET are not well established. Together with the traditional olivary model of ET (tremor should be generated by pacemaking neurons in the inferior olivary nucleus with rhythmic firing and an abnormal cerebellar output) recent evidences based in rigorous neuropathological studies suggested a cerebellar degenerative model with a partial loss of Purkinje cells, changes in Purkinje cell morphology, and alterations in connected neuronal populations.^32^

A recent study with magnetic resonance imaging T2∗-relaximetry, involving 24 ET patients and 25 age-matched healthy controls, found increased iron content in both globus pallidus, in both substantia nigra, and in the right dentate nucleus of the cerebellum of ET patients (although only bilateral pallidum remained significant after correction for multiple comparisons).^33^ To our knowledge, neither iron content or HMOX1 and HMOX2 expression have been measured in neuropathological studies of ET patients yet. It could be proposed that if the iron content should be increased in the cerebellum, HMOX should act as protective against iron-related oxidative stress, and alterations in *HMOX1* and *HMOX2* genes could be related with the cerebellar neurodegenerative model of the pathogenesis of ET.

Although the results of the present study should be taken with caution (a main limitation is the low sample size) and deserve further replication studies in other populations, they suggest a slightly decreased risk for ET in Spanish white individuals carrying the *HMOX1 rs2021746T* and *HMOX2 rs1051308G* allele variants.
